# Food Safety for Professionals (Second Edition)

**DOI:** 10.3201/eid0808.020287

**Published:** 2002-08

**Authors:** Camille Brewer, Arthur P. Liang

**Affiliations:** Food and Drug Administration, College Park, MD; and Centers for Disease Control and Prevention, Atlanta, GA

Drs. Cody and Kunkel have compiled an informative overview of food safety issues that is targeted toward dietetics professionals in particular but is also useful for food safety professionals. The guide contains many of the standard elements found in dietetics textbooks, including charts of toxic agents, information on specific foods and safety concerns, and basic food safety programs. The authors have wisely amended the standard textbook approach by including information on consumer needs and behaviors, a review of food safety surveillance programs, and a discussion of food safety laws and regulations. This edition includes additional chapters on suggestions for continuing education for dietetics professionals and an expanded list of resources, including online references.

This guide includes many useful details in a understandable format. The text is replete with tables (e.g., Descriptions of Specific Foodborne Bacterial Pathogens and Major Food Laws in the United States), which make the wealth of information easily readable. An extensive glossary specific to dietetic practice is included. The text also contains a continuing education self-assessment instrument for dietitians.

The breadth of the text is both its strength and weakness: a vast amount of material is covered, but inevitably, general statements are bound to leave out subtleties useful to the reader. In addition, foodborne illnesses caused by bacterial pathogens are emphasized; therefore, much of the discussion is focused on control measures for bacteria. The text incorrectly states, “…bacteria cause most of the cases of foodborne illness in the United States….” Most cases of foodborne illnesses are caused by unidentified agents. Of the illnesses of known origin, most are caused by viruses (1). On the other hand, the authors wisely include a discussion of parasites, an often overlooked as a cause of foodborne disease.

Similarly, the statement “FDA can order a product recall (or seize goods in the field)” on page 104 is inaccurate. While FDA can seize goods or request that a firm initiate recalls of food products, the agency's authority does not currently extend to mandatory recalls for most foods. FDA can, however, require a recall of infant formula under certain circumstances. The text would benefit from a deeper discussion of the role of the respective federal agencies in protecting the U.S. food supply. In addition, several important issues are not addressed or are not discussed thoroughly (e.g., global food safety considerations, the national food safety system, HACCP regulations for meat and poultry, seafood, and juice).

To their credit, the authors include a discussion of chronic sequelae of foodborne infections, an important area of consideration, particularly for dietitians.

 The text makes heavy use of Internet references. These references provide an abundance of current information but risk becoming out-of-date if the sites disappear or are not routinely updated.

Overall, the book is a valuable resource for its soup-to-nuts information approach. The book, a gold mine of useful information for dietitians, provides good one-stop-shopping for infectious disease scientists and professionals wishing to learn about the world of food safety.

## References

[1] Mead PS, Slutsker L, Dietz V, McCaig LF, Bresee JS, Shapiro C, Food-Related illness and death in the United States. Emerg Infect Dis. 1999;5:607–25. 10511517 10.3201/eid0505.990502 PMC2627714

